# Intranasal Volume Changes Caused by the Endoscopic Endonasal Transsphenoidal Approach and Their Effects on Nasal Functions

**DOI:** 10.1371/journal.pone.0151531

**Published:** 2016-03-24

**Authors:** Do Hyun Kim, Yong-Kil Hong, Sin-Soo Jeun, Yong Jin Park, Soo Whan Kim, Jin Hee Cho, Boo Young Kim, Sungwoo Han, Yong Joo Lee, Jae Hyung Hwang, Sung Won Kim

**Affiliations:** 1 Department of Otolaryngology-Head and Neck Surgery, Seoul St. Mary’s Hospital, College of Medicine, The Catholic University of Korea, Seoul, Korea; 2 Department of Neurosurgery, Seoul St. Mary’s Hospital, College of Medicine, The Catholic University of Korea, Seoul, Korea; Universita degli Studi di Palermo, ITALY

## Abstract

**Objective:**

We evaluated postoperative changes in nasal cavity volume and their effects on nasal function and symptoms after endoscopic endonasal transsphenoidal approach for antero-central skull base surgery.

**Study Design:**

Retrospective chart review at a tertiary referral center.

**Methods:**

We studied 92 patients who underwent binostril, four-hand, endoscopic endonasal transsphenoidal approach surgery using the bilateral modified nasoseptal rescue flap technique. Pre- and postoperative paranasal computed tomography and the Mimics^®^ program were used to assess nasal cavity volume changes at three sections. We also performed several pre- and postoperative tests, including the Connecticut Chemosensory Clinical Research Center test, Cross-Cultural Smell Identification Test, Nasal Obstruction Symptoms Evaluation, and Sino-Nasal Outcome Test-20. In addition, a visual analog scale was used to record subjective symptoms. We compared these data with the pre- and postoperative nasal cavity volumes.

**Results:**

Three-dimensional, objective increases in nasal passage volumes were evident between the inferior and middle turbinates (p<0.001) and between the superior turbinate and choana (p = 0.006) postoperatively. However, these did not correlate with subjectively assessed symptoms (NOSE, SNOT-20 and VAS; all nasal cavity areas; p≥0.05) or olfactory dysfunction (CCCRC and CCSIT test; all nasal cavity areas; p≥0.05).

**Conclusion:**

Skull base tumor surgery via an endoscopic endonasal transsphenoidal approach altered the patients’ nasal anatomy, but the changes in nasal cavity volumes did not affect nasal function or symptoms. These results will help surgeons to appropriately expose the surgical field during an endoscopic endonasal transsphenoidal approach.

## Introduction

The endoscopic endonasal transsphenoidal approach (EETSA) for cranial base surgery is the standard surgical method for sellar tumors[1,2]. The two-nostrils/four-hands technique was introduced to improve the manipulability of surgical instruments and increase the accessibility of the lateral sellar region[3]. Cerebrospinal fluid (CSF) leakage is an important complication after EETSA, although the incidence of CSF leakage decreased after Hadad *et al*.[4] introduced the nasoseptal flap concept[4,5]. For most sellar tumors, no intraoperative CSF leak is expected and the use of a nasoseptal flap may cause several complications, including nasal crusting, discharge, olfactory disorders, and longer operating times for complex cases[6,7]. Therefore, Rivera-Serrano *et al*.[8] introduced the nasoseptal “rescue” flap to reduce morbidity. This rescue flap can be harvested when sellar reconstruction is needed to close a resultant defect larger than expected or when there is unexpected CSF leakage. The technique consists of partially harvesting the superior and posterior ends of the flap to preserve its vascular pedicle and provides proper access to the sella during EETSA[8].

As EETSA has replaced a microscopic approach[1,2] and the number of EETSA surgeries featuring nasoseptal and nasoseptal rescue flaps has increased, the complications such as olfactory dysfunction after EETSA and postoperative quality of life have received attention[9,10]. Previously, we evaluated the change in olfactory function and nasal symptoms after EETSA[11,12]. In these studies, we evaluated that EETSA contribute to olfactory dysfunction and affect nasal symptoms independent of surgical type. So we decided to evaluate the changes of nasal function and symptoms after EETSA using scientific and objective method. Thus, we measured the change of nasal cavity volume and identified correlation between the change of nasal function and symptoms.

We used paranasal sinus computed tomography (PNS CT) and the Mimics^®^ program to reconstruct the postoperative three-dimensional (3D) changes in the nasal cavity. We explored the impact of such changes on nasal function and symptoms, using the Connecticut Chemosensory Clinical Research Center (CCCRC) test, Cross-Cultural Smell Identification Test (CCSIT), Nasal Obstruction Symptoms Evaluation (NOSE), Sino-Nasal Outcome Test-20 (SNOT-20), and a visual analog scale (VAS) assessing several nasal symptoms.

## Materials and Methods

This study was approved by the Institutional Review Board of Seoul St. Mary’s Hospital, and all individuals signed informed consent forms. Between November 2010 and November 2014, 331 patients with anterior and central skull base tumors underwent surgery via EETSA at Seoul St. Mary’s Hospital (Catholic University of Korea, Seoul, South Korea). As a retrospective study design, we excluded 47 patients with a history of sinonasal surgery, a history of olfactory disturbances before EETSA surgery, asthma, or sinonasal diseases. We also excluded 67 patients who did not undergo postoperative PNS CT and 78 patients who did not complete the pre- or postoperative CCCRC test, CCSIT, NOSE, SNOT-20, and VAS. The postoperative PNS CT and CCCRC test, CCSIT, NOSE, SNOT-20, and VAS scores were evaluated at least 6 months postoperatively. Moreover, 47 patients who did not undergo binostril four-hand EETSA with bilateral modified nasoseptal rescue flaps[13] were excluded. In this way, we ensured that all patients underwent identical surgeries. In this surgical method, the inferior, middle, and superior turbinates were preserved and lateralized, and posterior bony septum that included a portion of the perpendicular plate of the ethmoid bone, the vomer, and the anterior wall of the sphenoidal sinus was removed. Concomitant septoplasty was not done, and a posterior ethmoidectomy was included for wider sphenoid exposure[13]. Ultimately, the study enrolled 92 patients. All EETSA surgeries were performed by a single surgeon (S.W. Kim). After the approach procedure was finished, neurosurgery team of two surgeons performed tumor removal with binostril, four-hand technique.

Paranasal sinus CT was performed in the axial projection. During the evaluation, each patient lay supine on the scanner bed. Images were obtained at 120 kV and 180 mA over 7 s. Serial 0.6-mm-thick axial images were acquired and 1-mm-thick coronal images were reconstructed. All imaging was performed at the same original setting and Hounsfield unit (HU) (non-enhanced bone shadow; window, 2,000 HU; level, 400 HU).

**Fig 1:**
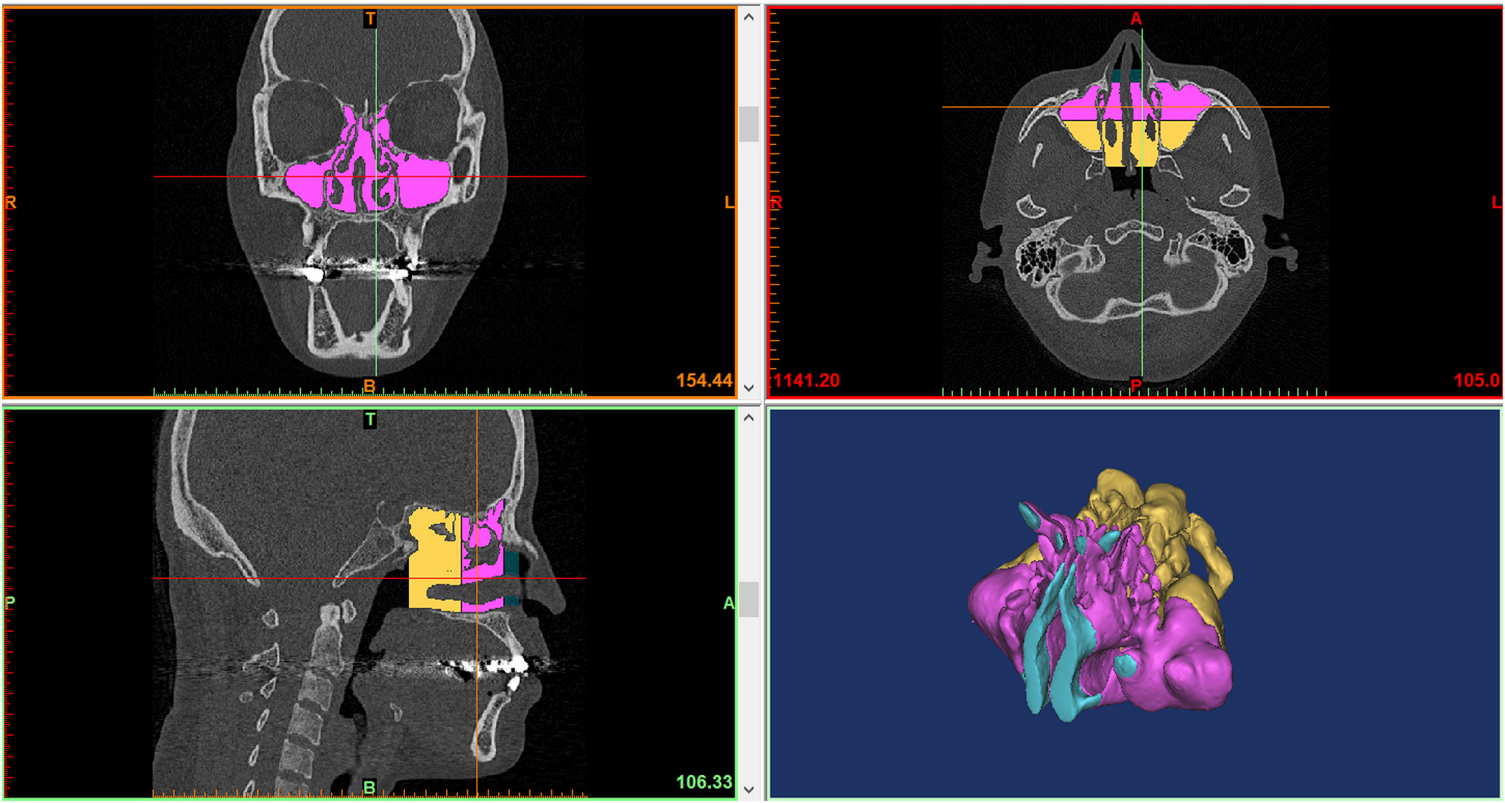
Nasal cavity volume measurement using Mimics^®^.

Three-dimensional reconstruction was performed using Mimics^®^. We divided the nasal cavity in three areas. The coronal boundary was defined at the level of the anterior end of the inferior (the nasal valve), middle, and superior turbinates and choana. The borders of the nasal cavity mucosa and air were determined automatically by the Mimics^®^ program using HU differences. The nasal cavity volume was calculated and reported in mm^3^ (Fig 1).

The CCCRC test used 1-butanol dissolved in mineral oil, as reported previously[14]. Commencing with a 1-butanol concentration of 4% (v/v) (dilution step 0), nine successive three-fold dilutions were prepared. The threshold was defined as the dilution at which the alcohol was identified in four consecutive trials. The CCSIT is a scratch-and-sniff test of twelve microencapsulated odorants with a forced choice of four alternatives per item[15]. Nasal symptoms were evaluated using the NOSE[16,17] and SNOT-20[18]; nasal stuffiness, sneezing, rhinorrhea, snoring, headache, facial pain, and olfactory changes were scored subjectively using a VAS (each score ranged from 0 to 10). Higher scores indicated more severe nasal symptoms.

All data are expressed as means ± standard deviation. The paired Student’s *t*-test was used to compare pre- and postoperative changes in the nasal cavity volume, and Pearson correlation coefficients were calculated to explore relationships between the nasal cavity volume changes and the CCCRC test, CCSIT, NOSE, SNOT-20, and VAS scores. A *p*-value <0.05 was considered to indicate statistical significance. All statistical analyses were conducted using SAS software (ver. 9.3; SAS Institute, Cary, NC, USA).

## Results

The study enrolled 92 patients, with a mean age of 47.1 years (range: 20–73 years); there were 51 (55%) males and 41 (45%) females. Table 1 shows types of tumors resected via EETSA. Pituitary macroadenoma were found in 77 patients (83.7%), Rathke's cleft cyst in 5 patients (5.4%), meningioma, chordoma and craniopharyngioma in 2 patients (2.2%), tuberculum sellae meningioma, carvenous sinus tumor, granular cell tumor of neurohypophysis and pseudocyst of pituitary gland in 1 patients (1.1%).

**Table 1:** Types of tumors resected via EETSA.

	Number of patients	Percentage (%)
Pituitary macroadenoma	77	83.7
Rathke's cleft cyst	5	5.4
Meningiomas	3	3.3
Chordoma	2	2.2
Craniopharyngioma	2	2.2
Carvenous sinus tumor	1	1.1
Granular cell tumor of neurohypophysis	1	1.1
Pseudocyst of pituitary gland	1	1.1

Postoperative volume changes in the nasal cavity were measured from the anterior end of the inferior turbinate to the middle turbinate (Area 1), from the middle turbinate to the superior turbinate (Area 2), and from the superior turbinate to the choana (Area 3) (Table 2). Increases in nasal volume after EETSA surgery were evident in Areas 1 (*p*<0.001) and 3 (*p* = 0.006), while no significant difference was evident in Area 2 (*p* = 0.801). The CCCRC test and CCSIT were used to evaluate pre- and postoperative olfactory function. No significant correlation between the CCCRC or CCSIT scores and nasal volume changes was evident in any area after EETSA surgery (Table 3). No significant correlation was seen between the pre- and postoperative NOSE and SNOT-20 scores and changes in volume within the nasal cavity (Table 4). We analyzed each VAS item in terms of the changes in nasal volume (Table 5). No significant correlation was evident between any change in volume in the three divided nasal cavity area and VAS items.

**Table 2:** Differences between the pre- and postoperative nasal cavity volumes.

	Preoperative		Postoperative		
	Mean (mm^3^)	SD	Mean (mm^3^)	SD	*p*-value
1. Inferior-middle turbinate	6054.78	4358.06	7008.93	4744.54	<.001*
2. Middle-superior turbinate	27178.62	11128.87	27385.39	9757.11	0.801
3. Superior turbinate-choana	27025.21	11064.44	29485.73	10621.32	0.006*

*P<0.05 for the test.

SD, standard deviation

**Table 3:** Correlation between CCCRC, CCSIT and nasal cavity volume changes.

	Score differences between pre and postoperative assessments			
	CCCRC		CCSIT	
	r	*p*-value	r	*p*-value
1. Inferior–middle turbinate	–0.004	0.968	0.024	0.818
2. Middle–superior turbinate	–0.07	0.505	–0.042	0.692
3. Superior turbinate–choana	0.009	0.929	0.108	0.304

CCCRC, Connecticut Chemosensory Clinical Research Center test; CCSIT, Cross-Cultural Smell Identification Test; r, Pearson correlation coefficient

**Table 4:** Correlation between the NOSE and SNOT-20 and nasal cavity volume changes.

	Score differences between the pre- and postoperative assessments			
	NOSE		SNOT-20	
	r	*p*-value	r	*p*-value
1. Inferior–middle turbinate	0.013	0.902	0.047	0.66
2. Middle–superior turbinate	0.123	0.244	0.059	0.576
3. Superior turbinate–choana	–0.108	0.306	–0.027	0.795

*P<0.05 for the test.

NOSE, Nasal Obstruction Symptoms Evaluation; SNOT-20, Sino-Nasal Outcome Test-20; r, Pearson correlation coefficient

**Table 5:** Correlation between VAS score and nasal cavity changes.

	Nasal cavity area		
Pre-post op. VAS item	1. Inf.–Middle	2. Middle–Sup.	3. Sup.–Choana
Q1			
r	–0.006	–0.098	0.056
*p*-value	0.952	0.351	0.596
Q2			
r	–0.059	–0.026	–0.016
*p*-value	0.579	0.808	0.883
Q3			
r	–0.148	–0.16	0.065
*p*-value	0.161	0.127	0.537
Q4			
r	0.026	0.082	–0.015
*p*-value	0.804	0.438	0.884
Q5			
r	–0.105	–0.064	–0.156
*p*-value	0.32	0.544	0.138
Q6			
r	0.003	0.005	–0.108
*p*-value	0.98	0.959	0.304
Q7			
r	0.062	0.025	–0.076
*p*-value	0.56	0.812	0.473

*P<0.05 for the test.

VAS, visual analog scale; Inf., inferior turbinate; Middle, middle turbinate; Sup., superior turbinate; Pre-post op., differences between pre- and postoperative score;

Q1, nasal stuffiness; Q2, sneezing; Q3, rhinorrhea; Q4, snoring, Q5, headache; Q6, facial pain; Q7, olfactory change; r, Pearson correlation coefficient

## Discussion

The EETSA to antero-central skull base tumors has reduced morbidity compared with previous surgical methods, while increasing the patient’s expectation of maintaining quality of life after surgery. The EETSA procedures include lateralization of the turbinates and a posterior ethmoidectomy for wider sphenoid exposure. A surgeon might hesitate if these widening procedures influenced postoperative complications. Decreased olfactory function is an important complication. The olfactory mucosa lies in the superior aspect of the nasal vault, overlying the superior nasal septum, cribriform plate, superior aspect of the superior turbinate, and medial surface of the middle turbinate[19]. However, the curvilinear incision using bilateral modified nasoseptal rescue flaps is remote from the olfactory neuroepithelium[13,20]. Therefore, anatomical volume changes in the nasal cavity caused by EETSA surgery, and their effects on olfactory function and other nasal symptoms, have attracted considerable interest.

Several reports have showed that an increase in nasal cavity volume is associated with a decrease in olfactory function[21–23]. In the context of changes to the nasal cavity, several explanations have been suggested. As the nasal cavity below the middle turbinate increases in size, inspired air is shunted away from the olfactory area, resulting in olfactory dysfunction[22]. As the size of nasal cavity reservoir is increased, more air is trapped, interrupting the flow of odorants toward the olfactory receptors; the airflow may be diverted from the olfactory region mechanically or local airflow changes may develop near the olfactory clefts[23]. Moreover, a posterosuperior septal defect after EETSA surgery can affect airflow dynamics in the region of the olfactory cleft[24]. Nasal volume change may affect humidity and the temperature of, and the shear stress on, the wall of the olfactory neuroepithelium[11,25,26]. Proper widening of the nasal cavity, including lateralization of the inferior, middle, or superior turbinates or posterior ethmoidectomy, is required when approaching skull base tumors varying in size and location. However, the influence of the degree of change in nasal cavity volume on olfaction has not been determined. We found an objective increase in the volume of the postoperative nasal passage between the inferior turbinate and middle turbinate and between the superior turbinate and choana. However, no correlation with subjective symptoms, including olfactory dysfunction, was evident. Binostril four-hand EETSA surgery using bilateral modified nasoseptal rescue flaps based on the anterior and middle skull base was developed to better expose the surgical field, reduce morbidity, and facilitate surgical instrument manipulation. Our study found that widening the surgical field was not associated with nasal functions or symptoms. In the future, we will examine postoperative changes and their effects on nasal function using computational fluid dynamics to measure airflow, temperature, and humidity.

Our study had several limitations and advantages. It enrolled a relatively large number of patients undergoing the same surgical procedure performed by a single surgeon, controlling the influence of surgical method and operator variables on outcomes. On the other hand, it also had some limitations. We examined nasal symptoms only once after surgery, at 6 months. In addition, the retrospective nature of the study renders the findings weaker than those afforded by randomized controlled studies. Also, most of our cases were pituitary tumors, and they might have lower rates of morbidity than the more expanded approaches required for tumors such as chordomas and meningiomas. However, the number of EETSA candidates with extensive tumors was too small to conduct sub-analysis investigating the relationship between the types of tumors and nasal symptoms. So, Future work should feature a larger group of patients with a longer follow-up period.

## Conclusions

Nasal cavity volumes increased after EETSA surgery, but these changes did not affect nasal function or symptoms. A wide, stable visual field and easy surgical instrument manipulation are important when performing surgery. This study confirmed that the surgical exposure using the bilateral modified nasoseptal rescue flap technique created during EETSA is suitable for antero-central skull base surgery.
